# Lysophosphatidic acid does not cause blood/lymphatic vessel plasticity in the rat mesentery culture model

**DOI:** 10.14814/phy2.12857

**Published:** 2016-07-08

**Authors:** Richard S. Sweat, Mohammad S. Azimi, Ariana D. Suarez‐Martinez, Prasad Katakam, Walter L. Murfee

**Affiliations:** ^1^ Department of Biomedical EngineeringTulane UniversityNew OrleansLouisiana; ^2^ Department of PharmacologyTulane UniversityNew OrleansLouisiana

**Keywords:** Angiogenesis, endothelial cell, lymphangiogenesis, lymphatic, lysophosphatidic acid, microcirculation

## Abstract

Understanding the mechanisms behind endothelial cell identity is crucial for the goal of manipulating microvascular networks. Lysophosphatidic acid (LPA) and serum stimulation have been suggested to induce a lymphatic identity in blood endothelial cells in vitro. The objective of this study was to determine if LPA or serum induces blood‐to‐lymphatic vessel phenotypic transition in microvascular networks. The rat mesentery culture model was used to observe the effect of stimulation on blood and lymphatic microvascular networks ex vivo. Vascularized mesenteric tissues were harvested from adult Wistar rats and cultured with LPA or 10% serum for up to 5 days. Tissues were then immunolabeled with PECAM to identify blood vessels and LYVE‐1 or Prox1 to identify lymphatic vessels. We show that while LPA caused capillary sprouting and increased vascular length density in adult microvascular networks, LPA did not cause a blood‐to‐lymphatic phenotypic transition. The results suggest that LPA is not sufficient to cause blood endothelial cells to adopt a lymphatic identity in adult microvascular networks. Similarly, serum stimulation caused robust angiogenesis and increased lymphatic/blood vessel connections, yet did not induce a blood‐to‐lymphatic phenotypic transition. Our study highlights an understudied area of lymphatic research and warrants future investigation into the mechanisms responsible for the maintenance of blood and lymphatic vessel identity.

## Introduction

From the design of microvascular‐based therapies to tissue engineering, a need exists to better understand the relationships between angiogenesis and lymphangiogenesis. Fundamental questions remain across scales. How is the patterning coordination between blood and lymphatic networks regulated? How do blood vessels connect to lymphatic vessels? Can blood endothelial cells become lymphatic endothelial cells? These types of questions highlight an emerging area of lymphatic research that focuses on endothelial cell identity and plasticity.

Regulating endothelial cell identity and fate are crucial for being able to manipulate microvascular networks (Oliver and Srinivasan 2010). Classically, cell differentiation was thought to be a terminal process. The discovery of induced pluripotency (Takahashi and Yamanaka 2006; Takahashi et al. 2007) emphasized the idea that a cell's fate was not irreversible and that a cell could be reprogrammed to switch phenotypes. This is evident in the blood vascular system with the regulation of arteriovenous endothelial cell identity (Adams 2003; Adams and Eichmann 2010). In the last ten to fifteen years, a handful of studies have also suggested that lymphatic endothelial cells can be plastic. For example, Johnson et al. showed that the loss of Prox1 causes endothelial cells to lose their lymphatic identity and adopt a blood endothelial cell phenotype resulting in blood‐to‐lymphatic perfusion (Johnson et al. 2008). Similar mispatterning outcomes have also been shown in mice lacking SLP‐76 (Abtahian et al. 2003), Rac1 (D'Amico et al. 2009), O‐glycan (Fu et al. 2008), or Fiaf (Backhed et al. 2007). These results, however, are largely based on developmental transgenic mice studies and fall short of identifying environmental factors that might be present in an adult scenario.

One candidate molecule is lysophosphatidic acid (LPA), which is a bioactive phospholipid present in most tissues throughout the body that acts through multiple G‐protein‐coupled receptors (GPCRs) to modulate cell proliferation, differentiation, and migration (Aoki et al. 2002). LPA has been linked to cardiovascular disease, neuropathy, neurological disorders, fibrosis, and cancer (Lin et al. 2010). It has also been reported to play an important role in controlling permeability and inflammatory properties of microvessels (Panchatcharam et al. 2014). During tumor progression, it is involved in numerous aspects of the disease, but one key factor is the promotion of angiogenesis (Mills and Moolenaar 2003). LPA has been shown to induce overexpression of VEGF (vascular endothelial growth factor), a potent stimulator of angiogenesis, in ovarian cancer cells. Additionally, Rivera‐Lopez et al. demonstrated the ability of LPA to directly stimulate angiogenesis in the chick CAM assay (Rivera‐Lopez et al. 2008). However, there is limited knowledge of the effect of LPA on lymphatic vessels. Recently, Mu et al. showed the ability of LPA to stimulate lymphangiogenesis in vitro, documenting increased cell proliferation, survival, migration, and tube formation (Mu et al. 2012). In the context of blood/lymphatic endothelial cell plasticity, Lin et al. in 2008 demonstrated that LPA upregulated the expression of both VEGF‐C and lymphatic marker expression by human umbilical vein endothelial cells (HUVECs) (Lin et al. 2008). To our knowledge, this is the first example of an exogenous factor that can induce a blood‐to‐lymphatic phenotypic transition. Supporting the potential for cell plasticity, Cooley et al. similarly showed in 2010 that serum stimulation of HUVECs in three‐dimensional culture also can induce a blood‐to‐lymphatic phenotypic transition (Cooley et al. 2010). The objective of this study was to determine if LPA or serum stimulation induces blood‐to‐lymphatic vessel phenotypic transition in microvascular networks. Using the rat mesentery culture model, which enables simultaneous observation of lymphatic and blood microvascular networks ex vivo (Stapor et al. 2013; Sweat et al. 2014; Azimi et al. 2015), we show that while LPA stimulation causes angiogenesis, it does not cause upregulation of lymphatic markers by blood vessels. Serum stimulation also did not cause blood endothelial cells along vessels to adopt a lymphatic identity. Our results offer a counterpoint to the published observations made in cell culture and emphasize the importance of microvascular network structure in the investigation of the mechanisms responsible for vessel identity.

## Materials and Methods

### Rat mesentery culture model

All experiments were performed in accordance with the guidelines of the Tulane University Institutional Animal Care and Use Committee. The rat mesentery culture model was used as previously described (Baluk et al. 2005; Sweat et al. 2014; Azimi et al. 2015). Briefly, adult male Wistar rats (Harlan, Indianapolis, IN) were anesthetized and euthanized, and then the mesentery was surgically exposed under aseptic conditions and mesenteric windows were harvested starting from the ileum. A mesenteric window was defined as the thin, translucent connective tissue between artery/vein pairs that feed the small intestine. Tissues were rinsed in DPBS (Dulbecco's phosphate buffered saline) (Cat. No. 14040‐133; Life Technologies, Carlsbad, CA) and cultured at 37°C in 12‐well culture plates with serum‐free minimum essential media (MEM) (Cat. No. 11095‐080; Life Technologies) supplemented with penicillin/streptomycin (Cat. No. 15140‐122; Life Technologies) with or without LPA (Cat. No. L7260; Sigma‐Aldrich, St. Louis, MO) changed daily for 3 or 5 days. Additional tissues were cultured for 7 and 9 days in media supplemented with 20 and 100 μmol/L LPA. Additional tissues were also cultured in MEM supplemented with 10% fetal bovine serum (FBS) (Cat. No. 16000‐044; Life Technologies) for 5 days. FBS was used to match the serum stimulus in the Cooley et al., 2010 study, which demonstrated an effect of FBS on HUVEC blood‐to‐lymphatic vessel transition (Cooley et al. 2010). In the rat mesentery culture model, FBS has also been shown to cause a robust remodeling response (Azimi et al. 2015).

### Immunohistochemistry

Cultured tissues were rinsed in PBS (phosphate buffered saline), mounted on glass slides, and fixed in 100% methanol at −20°C for 30 min. Tissues were washed in PBS + 0.1% saponin (Cat. No. 57900‐100G, Sigma‐Aldrich) three times for 10 min each then immunolabeled with primary antibodies and fluorescently labeled secondaries. Blood vessels were identified with a mouse monoclonal biotinylated antibody against PECAM (platelet endothelial cell adhesion molecule) (1:200, Cat. No. 555026, BD Pharmingen, San Diego, CA) followed by a Cy3‐conjugated streptavidin (1:500, Cat. No. 016‐160‐084; Jackson Immunoresearch, West Grove, PA). Lymphatic vessels were identified with either a rabbit polyclonal antibody against LYVE‐1 (1:100, Cat. No. 11‐034; AngioBio, Del Mar, CA) along with an Alexa Fluor 488‐conjugated goat anti‐rabbit antibody (1:100, Cat. No. 111‐545‐144; Jackson Immunoresearch), or a mouse monoclonal antibody against Prox1 (1:100, Cat. No. NB600‐1001; Novus Biologicals, Littleton, CO) along with an Alexa Fluor 594‐conjugated goat anti‐mouse antibody (1:100, Cat. No. 115‐586‐072; Jackson Immunoresearch). All antibodies were diluted in PBS + 0.1% saponin + 2% bovine serum albumin and incubated at room temperature for 1 h.

### Quantification of lymphangiogenesis and angiogenesis

Tissues were randomly selected for quantification of blood capillary sprouts (*n* = 7–8 tissues per group from 4 rats) and lymphatic sprouts (*n* = 8 tissues per group from 4 rats). The entire blood and lymphatic vascular area of each tissue was imaged at 4× magnification, where vascular area was defined as the area circumscribed by the sum of each individual network within a tissue. The number of blood capillary sprouts, lymphatic sprouts, and blood vessel length were quantified from five randomly selected 4× fields of view and the average values were normalized to the average vascular area per image. Blood and lymphatic vessels were identified by PECAM and LYVE‐1 expression, respectively. Blood capillary sprouts were defined as blind‐ended blood vessel segments extending from existing vessels. Lymphatic sprouts were defined as LYVE‐1‐positive endothelial cell extensions off a host vessel. They were differentiated from typical lymphatic blind ends, which are the terminal, blunt‐ended vessels associated with a functional initial lymphatic network (Baluk et al. 2005; Sweat et al. 2012), based on a diameter and morphology similar to blood capillary sprouts. Filopodia are short pseudopod projections that are typically less than the diameter of the host vessel. In contrast, lymphatic sprouts exhibited a length longer than the host diameter. Lymphatic filopodia were quantified from three randomly selected 10× fields of view per tissue (*n* = 7 tissues per group from 4 rats per group) and normalized to the total lymphatic vessel length per image.

### Imaging

Images were acquired using an Olympus IX70 inverted microscope (Olympus America, Center Valley, PA) with 4× dry, 10× dry, 20× oil, and 60× oil objectives and coupled to a Photometrics CoolSNAP EZ camera (Photometrics, Tucson, AZ). Image analysis and quantification was done using ImageJ 1.49o (U.S. National Institutes of Health, Bethesda, MD). Confocal microscopy images of lymphatic/blood endothelial cell connections were captured with a Zeiss LSM 7 Live high‐speed laser confocal microscope equipped with 488 and 532 diode laser lines (Carl Zeiss MicroImaging) and a C‐Apochromat 63× (NA = 1.20 W Korr M27) objective. For Figure 5, confocal projections were obtained by stacking multiple 0.9‐*μ*m‐thick optical sections.

### Statistical analysis

Comparisons of angiogenesis metrics were made using Student's *t*‐tests to compare experimental groups to control groups. Results were considered statistically significant when *P* < 0.05. Statistical analysis was performed using SigmaStat ver. 3.5 (Systat Software, San Jose, CA). Values are presented as means ± standard error of the means.

## Results

### LPA stimulated angiogenesis in ex vivo microvascular networks

**Figure 1:**
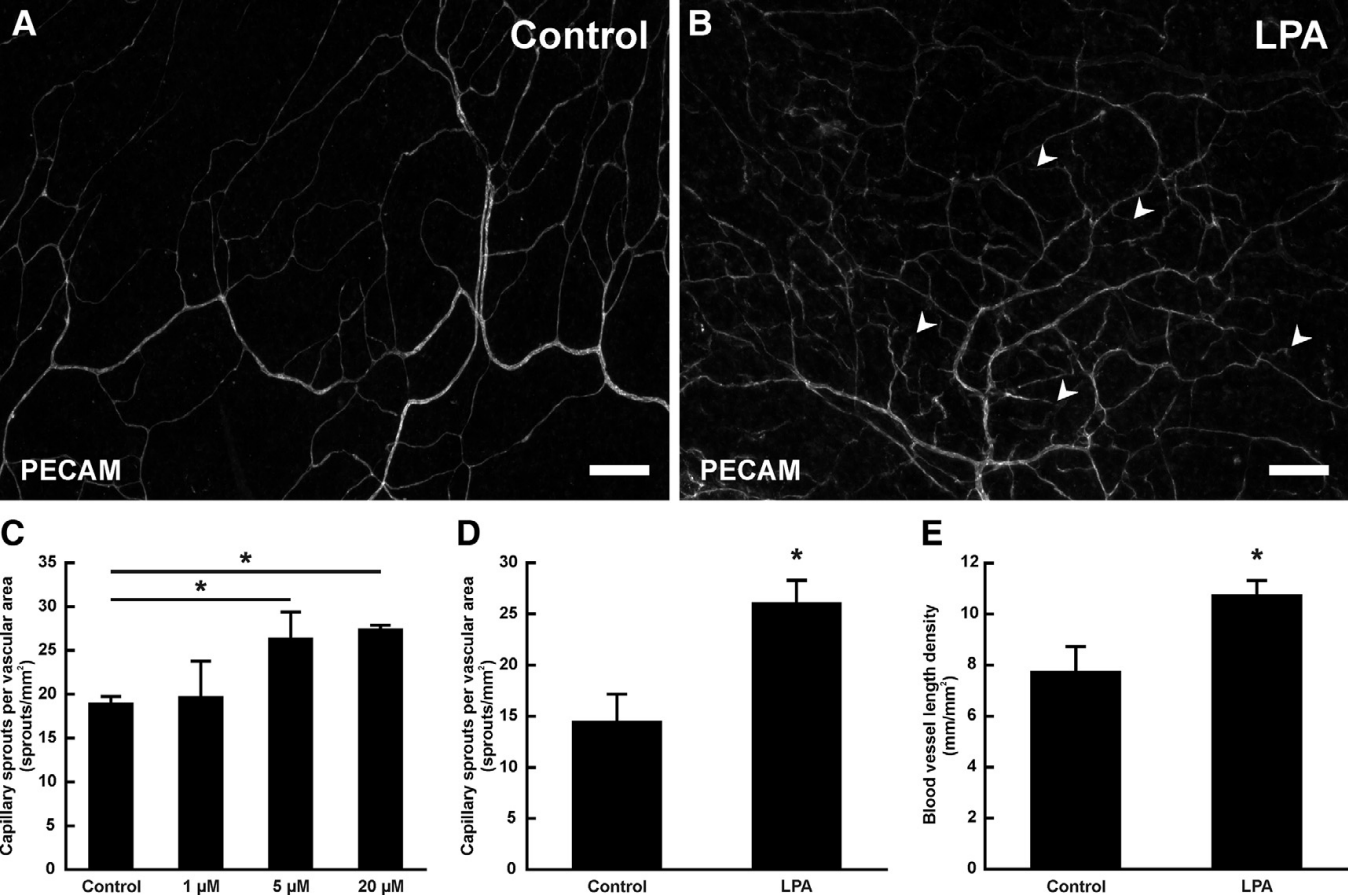
LPA stimulation of angiogenesis in the rat mesentery culture model. (A) Representative image from control group cultured with MEM‐only for 3 days. (B) Representative image from tissues cultured with 20 μmol/L LPA for 3 days. Blood vessel networks cultured with LPA showed increased vessel density and more capillary sprouts (arrowheads). Scale bars = 200 *μ*m. (C) Quantification of the number of blood capillary sprouts per vascular area after treatment with various concentrations of LPA for 3 days. (D) The number of blood capillary sprouts per vascular area was increased after 3 days with 20 μmol/L LPA. (E) Blood vascular length density was increased after 3 days cultured with 20 μmol/L LPA. *Significant difference compared to control group (*P* < 0.05).

Microvascular networks in tissues cultured with LPA showed evidence of increased angiogenesis (Fig. 1A and B). After 3 days of culture, blood vascular networks exhibited an apparent increase in the number of blind‐ended capillary sprouts and an increase in the density of the network. The concentration of LPA was selected based on values previously reported in the literature (Lin et al. 2008; Rivera‐Lopez et al. 2008; Mu et al. 2012) and experimental capillary sprouting response data (Fig. 1C). The quantity of 20 μmol/L LPA showed a consistent, robust angiogenic sprouting response compared to lower doses of LPA and MEM controls and therefore was chosen for subsequent experiments. Quantification of angiogenic metrics indicated significant increases in the number of capillary sprouts per vascular area (*P* = 0.005; Fig. 1D) and vascular length density (*P* = 0.02; Fig. 1E) in tissues cultured with 20 μmol/L LPA compared with MEM‐only control tissues.

### LPA effect on lymphangiogenesis

**Figure 2:**
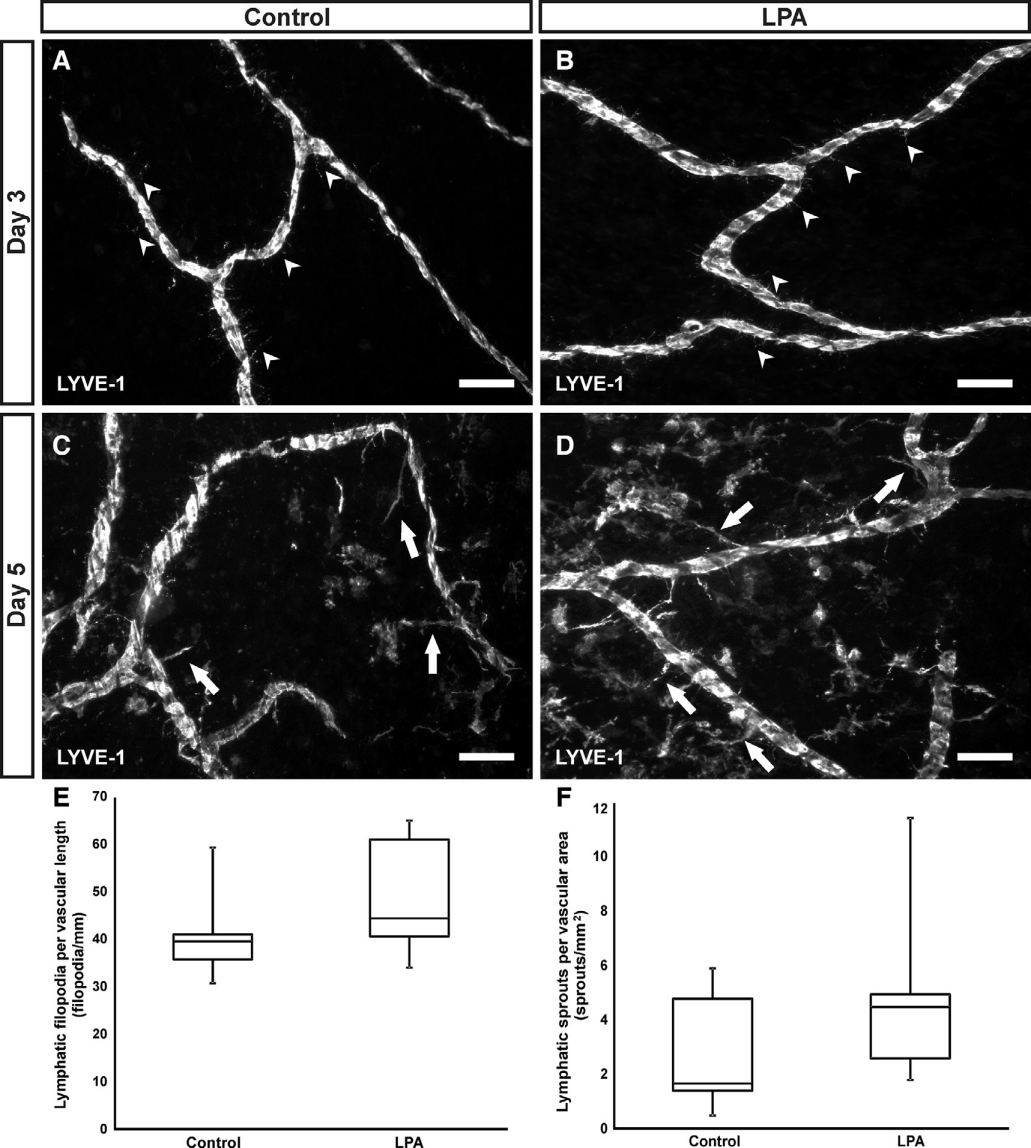
LPA effects on lymphangiogenesis in ex vivo microvascular networks. (A, B) Representative images from control and 20 μmol/L LPA tissues cultured for 3 days. Lymphatic vessels exhibited limited filopodia formation (arrowheads). (C, D) Representative images from control and 20 μmol/L LPA tissues cultured for 5 days. Arrows identify lymphatic sprouts. (E) Box‐and‐whisker plots of the day 3 data for the number of lymphatic filopodia per vascular length for control and LPA groups. (F) Box‐and‐whisker plots of the day 5 data for the number of lymphatic sprouts per vascular area for control and LPA groups. Scale bars = 100 *μ*m.

After 3 days of culture with 20 μmol/L LPA, there was no apparent lymphatic sprouting. Examples of increased filopodia were observed in some tissues after 3 days for both the MEM‐only control and LPA stimulated tissues (Fig. 2A and B). As documented previously (Sweat et al. 2012, 2014), lymphangiogenesis commonly occurs at a later time point than angiogenesis. Therefore, lymphangiogenesis was also assessed in tissues cultured for 5 days. Examples of lymphatic vessel sprouts were observed in a subset of stimulated tissues following culture for 5 days with 20 μmol/L LPA (Fig. 2B and C). The mean levels of lymphatic filopodia and sprouting for the LPA stimulated groups was greater than the control group (Fig. 2E and F). However, the power for the 3 and 5 day lymphatic growth metrics were 0.29 and 0.15, respectively. Based on these low power analysis values (<0.8), a statistical comparison was not performed. For a 0.8 power value, the samples sizes would need to be 25 per 3 day group and 65 per 5 day group. LYVE‐1 labeling of cultured tissues also identified interstitial cells, which we have previously characterized as including CD11b‐positive macrophages (Sweat et al. 2012).

### Blood and lymphatic vessel identity is maintained in LPA stimulated microvascular networks

**Figure 3:**
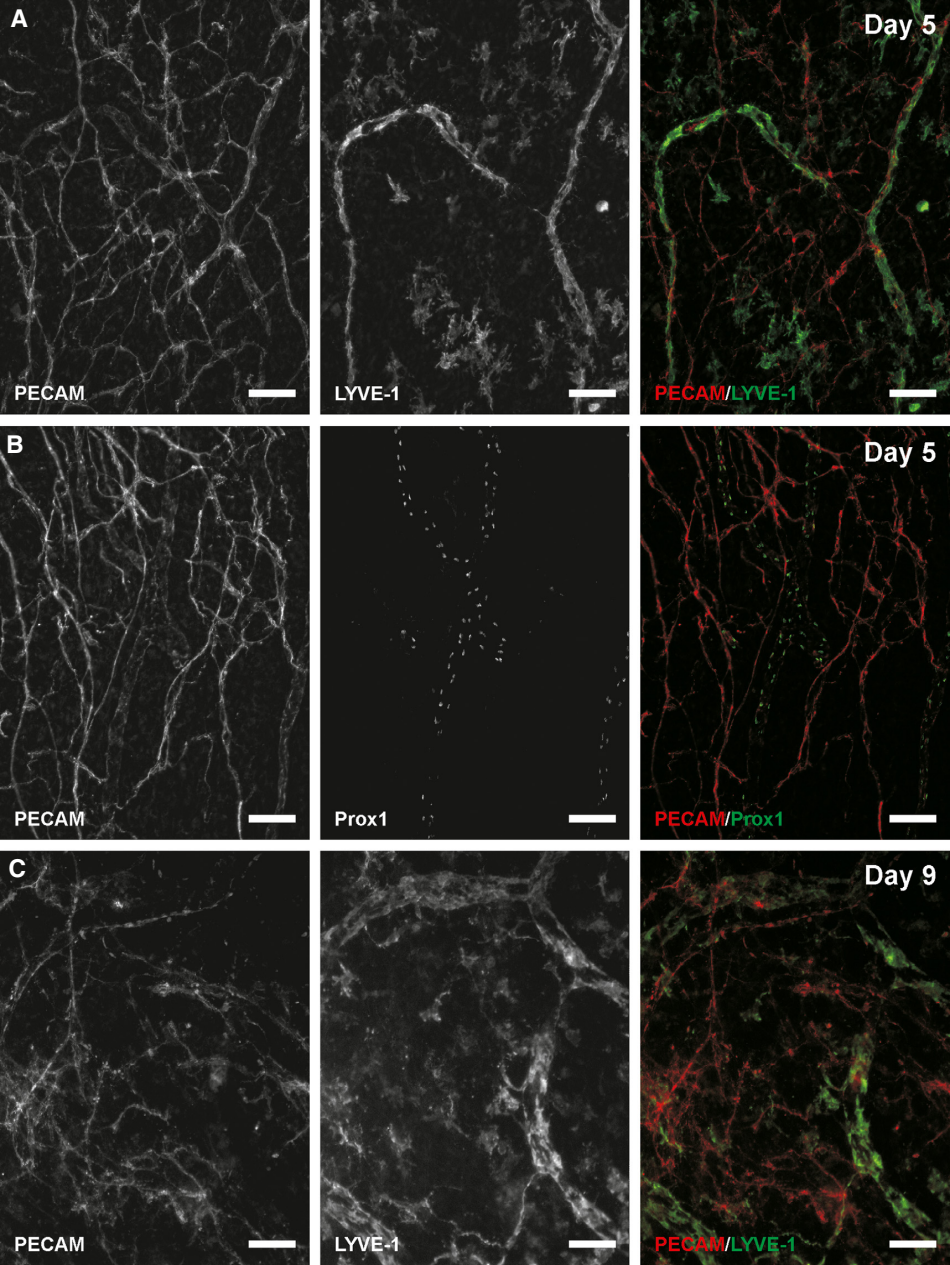
Lymphatic marker expression after 20 μmol/L LPA stimulation. (A) On day 5, LYVE‐1 expression was restricted to vessels that possessed a morphology typical of preexisting lymphatic vessels. (B) On day 5, Prox1‐positive nuclei were similarly only observed along these typical lymphatic vessels and not blood vessels. (C) Representative image of a network cultured for 9 days. PECAM‐positive blood capillaries lacked positive LYVE‐1 labeling. Scale bars = 100 *μ*m.

**Figure 4:**
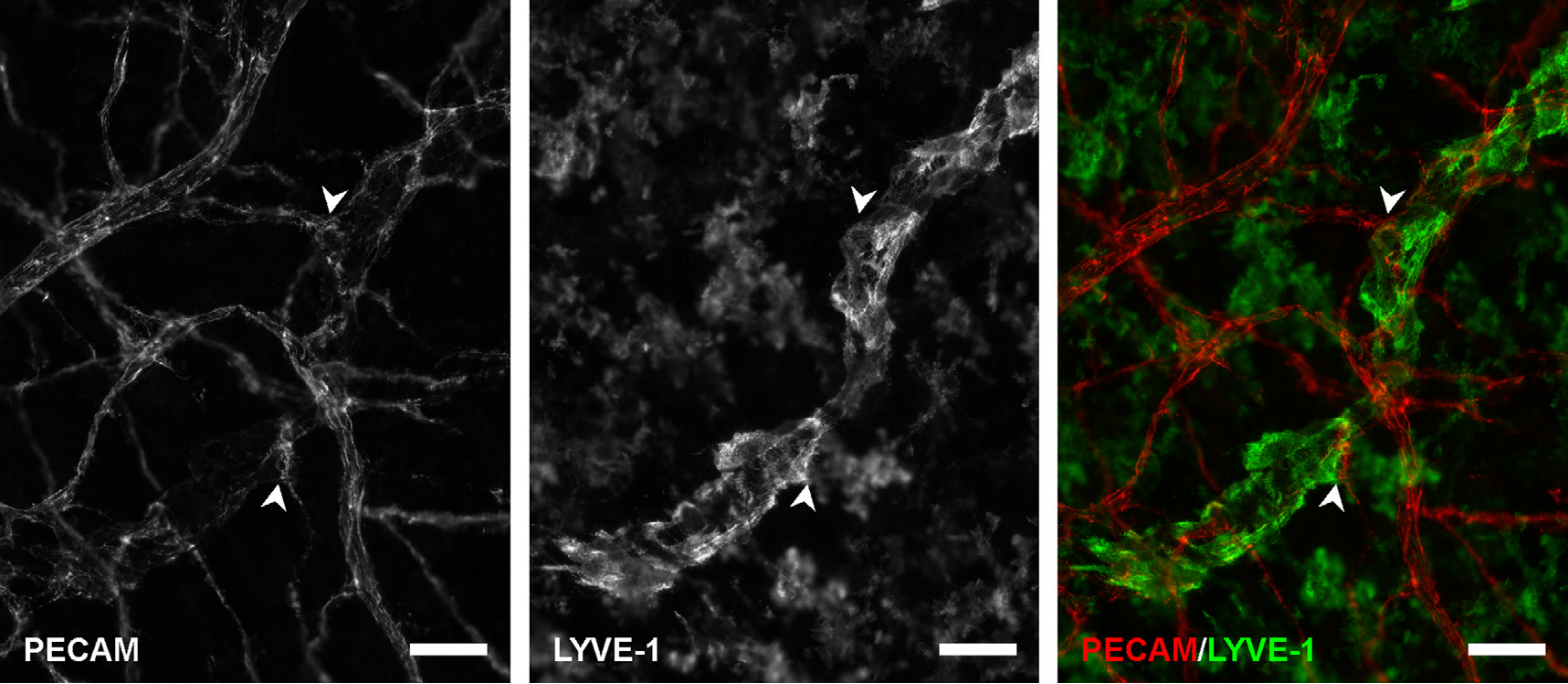
Example of blood/lymphatic vessel connections at day 3 after stimulation with LPA. LYVE‐1 labeling identified lymphatic endothelial cells at PECAM‐positive connection sites (arrowheads). These apparent connections appeared similar to those that have been previously confirmed to have continuous PECAM junctional labeling by endothelial cells across connection sites by submicrometer optical sections using confocal microscopy (Phamduy et al. 2015; Robichaux et al. 2010; Sweat et al. 2014). LYVE‐1 expression was restricted to the existing lymphatic vessel. Scale bars = 50 *μ*m.

Microvascular networks in tissues stimulated by LPA were evaluated for evidence of blood/lymphatic endothelial cell plasticity. Endothelial cells were identified as blood or lymphatic based on phenotypic marker expression, cell location, and vessel morphology consistent with previously established methodologies (Sweat et al. 2012, 2014; Stapor et al. 2013). Blood endothelial cells did not exhibit evidence of adopting a lymphatic phenotype after stimulation with 20 μmol/L LPA for 3 or 5 days. The endothelial cells of apparent blood vessels maintained a relatively more intense expression of PECAM, did not express LYVE‐1 or Prox1, and maintained the vessel morphology appropriate for their location along the blood vascular network hierarchy. Correspondingly, expression of LYVE‐1 and Prox1 was restricted to vessels that displayed typical lymphatic vessel traits, including reduced PECAM expression, increased vessel diameters, and irregular vessel morphologies (Fig. 3). To determine whether blood‐to‐lymphatic vessel transition could be induced at higher concentrations and longer durations, additional tissues were stimulated with 20 μmol/L LPA for 7 and 9 days and with 100 μmol/L LPA for 7 and 9 days. Qualitative observations (Fig. 3C) support the maintained LYVE‐1 negative blood vessel identity. Even at sites of apparent physical connections between lymphatic and blood capillaries, which we have previously defined by PECAM‐positive junctional continuity (Robichaux et al. 2010; Sweat et al. 2012), no change in phenotype was detected supporting the maintained separation of blood and lymphatic vessel identity at the cell–cell level (Fig. 4).

### Blood/lymphatic endothelial cell dynamics in serum‐stimulated microvascular networks

**Figure 5:**
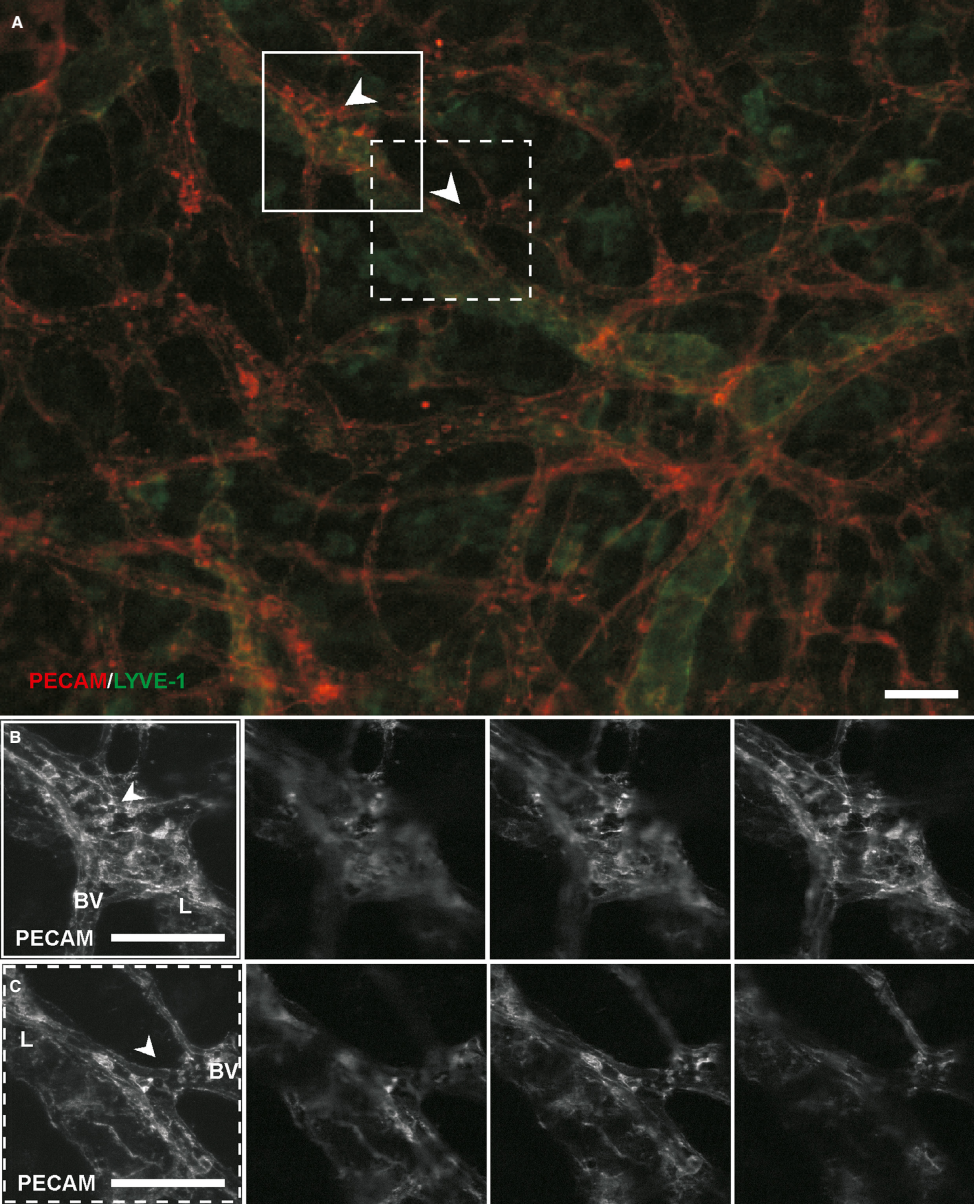
Blood/lymphatic vessel mispatterning in serum‐stimulated microvascular networks. (A) Example of a microvascular network region on Day 5 post tissue stimulation with 10% serum. PECAM labeling identifies both blood and lymphatic endothelial cells. LYVE‐1 labeling identifies lymphatic endothelial cells. At connections sites (boxes), LYVE‐1 negative cell (arrowhead) from the blood vessel side connects to a LYVE‐1 positive lymphatic vessel. (B, C) Confocal projections of sub‐micron optical sections for the connection sites. A subset of the optical sections are shown to the right per projection. PECAM labeling of endothelial junctions was continuous from blood vessels (BVs) to lymphatic vessels (L). Scale bars = 50 *μ*m.

**Figure 6:**
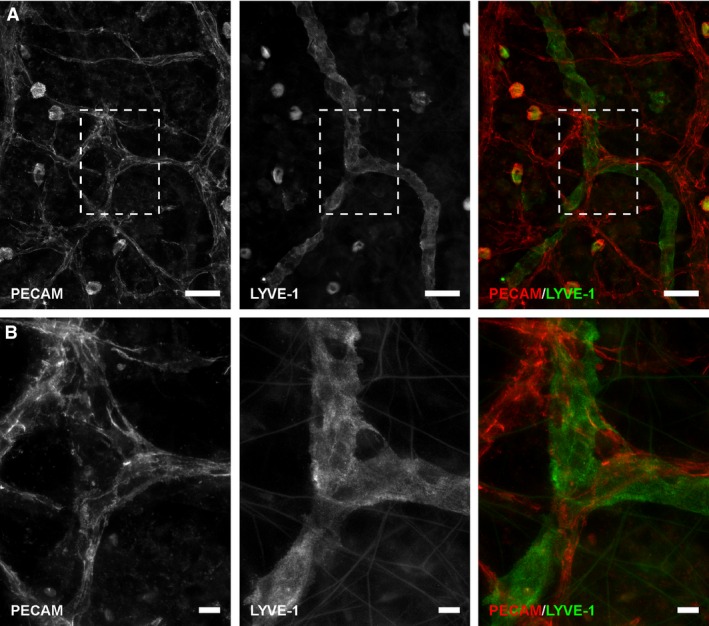
Blood/lymphatic endothelial cell phenotypic identity is maintained at connection sites. (A) Example of a microvascular network region after 5 days of stimulation with 10% serum. PECAM labeling identifies both blood and lymphatic endothelial cells. LYVE‐1 labeling identifies lymphatic endothelial cells. At connection sites (inset; B), LYVE‐1^−^ cells from the blood capillary sprout connect to LYVE‐1^+^ lymphatic vessels. Scale bars = (A) 50 *μ*m, (B) 10 *μ*m.

Serum was used as an alternative stimulus based on the report by Cooley et al. 2010 showing that serum also induces a blood‐to‐lymphatic phenotype transition in three‐dimensionally cultured HUVECs (Cooley et al. 2010). Additional microvascular networks were stimulated with 10% FBS in the rat mesentery culture model for 5 days based on our previous observation that serum caused a robust microvascular network remodeling response (Azimi et al. 2015). Stimulated networks typically displayed an apparent plexus of blood and lymphatic vessels, which, in contrast to the LPA stimulated vessels, were difficult to distinguish based on PECAM morphology alone. Remodeled networks also typically displayed numerous apparent blood/lymphatic endothelial cell connections suggestive of vessel integration (Fig. 5). Blood/lymphatic endothelial cell connections were identified by the observation of PECAM^+^/LYVE‐1^−^ blood endothelial cells apparently merging with PECAM^+^/LYVE‐1^+^ lymphatic endothelial cells (Fig. 5). Identification of apparent vessel connections is consistent with our previous identification and characterization of connections in mesentery tissue with confocal microscopy of junctional continuity in submicron optical sections (Sweat et al. 2012, 2014; Stapor et al. 2013) and confirmed in this study by continuous PECAM labeling across both vessel types using similar confocal methods (Fig. 5B and C). At connection sites, endothelial cells maintained their distinct blood or lymphatic identity, based on the discrete expression of LYVE‐1 along the lymphatic vessel and not along the blood capillary (Fig. 6).

## Discussion

The primary finding of this study is that lysophosphatidic acid (LPA) and serum stimulation was not sufficient to induce blood‐to‐lymphatic phenotypic transition. These results emphasize the importance of investigating blood/lymphatic vessel relationships in the context of real microvascular networks and motivate new studies to identify mechanisms responsible for the maintenance of vessel identity in adult tissues.

The rat mesentery culture model was selected for this study because it offers a unique capability of investigating ex vivo blood and lymphatic vessel dynamics simultaneously in the same microvascular network (Stapor et al. 2013; Sweat et al. 2014) and while we have not previously reported lymphatic‐to‐blood vessel plasticity in this model, our laboratory has demonstrated that the model can be used to investigate (1) angiogenesis (Stapor et al. 2013; Sweat et al. 2014; Azimi et al. 2015; Phamduy et al. 2015); (2) pericyte‐endothelial cell interactions (Stapor et al. 2013); (3) lymphangiogenesis (Sweat et al. 2014); and (4) the formation of blood/lymphatic endothelial cell connections (Stapor et al. 2013). Vascular remodeling in these previous studies was induced via serum, bFGF, VEGF‐A, VEGF‐C, and cancer cell stimulation. Specifically, in our Sweat et al., 2014 study we demonstrated that VEGF‐C stimulation caused lymphangiogenesis and angiogenesis (Sweat et al., 2014) and in our Stapor et al., 2013 study we reported observations of increased lymphatic filopodia stimulation in response to bFGF supplemented media (Stapor et al. 2013). Altogether our previous results support the use of the model for investigating unknown effects of a physiological agent, such as LPA as in the case of this study, on blood and lymphatic vessels. Moreover, an effect of LPA on vascular remodeling in the rat mesentery culture model is supported by our data showing LPA media supplementation causes increased angiogenesis. Another advantage of the model is the thinness of the tissue, which allows *en face* observation across the hierarchy of microvascular networks down to the single cell level. This advantage is important for the immunohistochemical determination of cell phenotype changes and has been leveraged in previous work to identify novel angiogenic pericyte phenotypes (Murfee et al. 2006; Stapor and Murfee 2012) and lymphangiogenic endothelial cell phenotypes (Sweat et al. 2012) over similar time courses as this study. The ability to detect phenotypic changes using immunohistochemistry is also supported by the observation of interstitial cell upregulation of LYVE‐1 in LPA tissues after culture, which is consistent with our previous in vivo characterization of LYVE‐1 upregulation by interstitial cells, including CD11b‐positive macrophages, in the same tissue during an inflammatory response (Sweat et al. 2012). A current limitation of the rat mesentery culture model is the lack of flow, which is known to be important for regulating endothelial cell identity. However, this limitation also exists in most two‐ and three‐dimensional cell culture experiments, which have been used to show lymphatic/blood endothelial cell plasticity (Lin et al. 2008; Cooley et al. 2010). Thus, we speculate that our model provides a network scenario that can be compared to those single cell assays and used to isolate network organization effects independent of flow.

An intriguing recent study in the area of blood/lymphatic plasticity by Lin et al. demonstrated that LPA upregulated lymphatic marker expression in HUVECs in vitro within 24 h (Lin et al. 2008). This ability for blood‐to‐lymphatic phenotypic transition is also supported in work by Cooley et al. showing that serum stimulation of HUVECs over 3 days in vitro can similarly cause lymphatic marker upregulation (Cooley et al. 2010) and evidence by Keushchnigg et al. documenting a hybrid blood‐lymphatic phenotype in HMEC‐1 (human microvascular endothelial cell) and TIME (telomerase‐immortalized human microvascular endothelium cell) endothelial cells lines (Keuschnigg et al. 2013). Collectively, these studies suggest endothelial cells possess an innate plasticity. These experiments, however, do not resolve questions about whether this property holds true in pre‐existing, adult microvascular networks. Interestingly, the study published by Lin et al. does provide images of lymphatic marker positive structures in sections from a Matrigel plug loaded with LPA 7 days after transplantation (Lin et al. 2008), yet whether this observation was explained by the phenotypic transition of pre‐existing blood vessels or ingrowth of new lymphatic vessels remains to be determined. Our results using the rat mesentery culture model suggest that LPA stimulation alone for 3 and 5 days is not sufficient to cause blood endothelial cells associated with intact vessels to adopt a lymphatic phenotype. While future studies might be needed with different concentrations and exposure durations to optimize our study, it is important to note that stimulation with 20 or 100 μmol/L LPA for 7 or 9 days also was not sufficient to cause blood vessels to label for LYVE‐1.

We speculate that scenario differences are a possible explanation for the discrepancy in our ex vivo network versus the published in vitro results. Correspondingly, the 3 day, 20 μmol/L LPA stimulation was also repeated in a subset of tissues that had been previously cultured for 7 days with MEM‐only (data not shown) to further separate the microvascular networks from their in vivo conditions and invoke a more in vitro scenario. No evidence of lymphatic phenotype was again observed in blood vessels. Another explanation might be that different concentration thresholds are needed in our model versus two‐dimensional culture. The concentration of LPA used in this study exceeds that which was previously shown to stimulate plasticity in vitro and produced a consistent, significant angiogenic response, indicating sufficient vascular stimulation. Additional tissues cultured with the fivefold higher concentration of 100 μmol/L LPA for 3, 7 or 9 days (data not shown) also did not produce a phenotypic change, confirming the concentrations used should have been sufficient to elicit a vascular response. Using serum as an alternative stimulus, we further show that lack of vessel plasticity response is not LPA specific supporting the potential importance of the intact microvascular network environment.

While endothelial responsiveness in the rat mesentery culture model is supported by our data showing LPA media supplementation causes increased angiogenesis, the lack of effect on blood‐to‐lymphatic endothelial cell transition might be explained by the unknown kinetics of LPA exchange, LPA availability, LPA sensitivity influenced by inhibitor of LPA‐mediated responsiveness (Senda et al. 2009) and regulatory enzymes, like lipid‐phosphate phosphatase (LPP)‐3 (Park and Kazlauskas 2012). The work by Park and Kazlauskas suggest that endothelial cells have the capacity to degrade LPA in specific growth factor environments, and Senda et al. demonstrated that LPP3 activity inhibition induces capillary formation in lymphatic endothelial cells indicating that the presence of this enzyme also plays a role in mediating LPA effects (Senda et al. 2009). Future investigations are needed to fully characterize local environmental LPA processing and to evaluate whether these local dynamics are altered in an intact microvascular network model scenario versus a single cell culture assay.

The main contribution of our study is that LPA or serum induction of blood‐to‐lymphatic endothelial cell phenotypic transition was not observed in the rat mesentery culture model. This result counters the results shown with similar LPA (Lin et al. 2008) or serum (Cooley et al. 2010) stimuli in single cell culture assays. In the context of the emerging area of lymphatic/blood endothelial cell plasticity, our study highlights the potential differences in signaling regulation between these single cell systems and more complex scenarios. Other possible explanations for our different results could be human versus rodent models and male versus female effects. Future investigations will also be needed to determine if rodent derived endothelial cell lines exhibit a blood‐to‐lymphatic phenotypic transition in vitro similar to HUVECs or if vessel plasticity could be induced via LPA stimulation in rat mesenteric microvascular networks from female rats.

We hypothesize that a possible explanation for the maintenance of blood vessel identity in the rat mesentery culture system might also be vascular pericytes. Cooley et al. showed that blood endothelial cells can undergo transdifferentiation to a lymphatic phenotype when cultured in a 3‐D collagen gel in vitro and stimulated with serum (Cooley et al. 2010). However, this phenomenon was inhibited when the endothelial cells were cocultured with perivascular cells. Interestingly in our current study, blood vessel versus lymphatic identity was maintained in the cultured rat mesentery tissues stimulated with 20% serum in spite of increased connections across the systems indicative of mispatterning (Fig. 5). The rat mesentery is known to have pericytes present along blood vessels and they have been confirmed to survive and maintain their perivascular location during culture (Stapor et al. 2013). Furthermore, evidence, suggested by Motiejūnait≐ et al., that pericytes counteract the influence of LPA provides additional support for pericyte‐mediated maintenance of endothelial cell identity (Motiejunaite et al. 2014). They demonstrated that pericytes rapidly metabolized LPA with and without the presence of endothelial cells and, consequently, protected endothelial cells tubes from degradation. This result led to the hypothesis that the protective effect could be due to upregulation of LPPs. This mechanism, however, has yet to be confirmed in vivo (Motiejunaite et al. 2014). We feel these examples supporting a role for pericytes in regulating endothelial cell dynamics underscore the necessity of investigating these relationships between systems in real microvascular networks and that the rat mesentery culture model is a promising method to help link in vitro phenomena to in vivo physiology, warranting future studies targeting pericyte‐mediated effects on endothelial cell fate.

Our results showing that LPA promotes angiogenesis concur with previous studies that demonstrated its angiogenic effect. LPA is known to stimulate endothelial cell migration and proliferation in vitro (English et al. 1999; Lee et al. 2000). Rivera‐Lopez et al. demonstrated that a robust angiogenic response similar to VEGF stimulation was mediated by the LPA_3_ and possibly LPA_1_ receptor when using the chicken chorioallantoic membrane (CAM) assay (Rivera‐Lopez et al. 2008). Support for an effect of LPA on lymphangiogenesis in vitro is provided by Mu et al. where their work demonstrated that LPA promoted lymphatic endothelial cell proliferation, migration, and tube formation via LPA_2_ receptor (Mu et al. 2012) signaling, as opposed to LPA_1_ and LPA_3_ receptors previously discussed to be involved in LPA‐induced angiogenesis (Rivera‐Lopez et al. 2008). Hence, potential differential effects of LPA on blood vessels versus lymphatics might be explained by vessel type specific LPA receptor expression. The direct effect of LPA on adult lymphangiogenesis in vivo, however, has not yet been demonstrated. The trend associated with our lymphangiogenesis data suggests the potential for tissue specific responses and motivates additional studies to investigate how network‐specific characteristics might influence local LPA effects or whether lymphangiogenesis can be induced with higher LPA concentrations or with longer exposure times. To this end, the additional tissues treated with 100 μmol/L LPA for 7 and 9 days displayed examples of localized lymphatic sprouting, yet this effect was again not uniform across a network. Still, future studies are needed to confirm an effect of LPA on lymphangiogenesis and to determine whether possible differences in LPA receptor expression patterns along blood vessels versus lymphatic vessels might explain possible vessel type specific responses.

## Conclusions

We investigated the effects of LPA, a molecule shown to trigger blood‐to‐lymphatic endothelial cell plasticity in two‐dimensional cell culture (Lin et al. 2008), on microvascular networks. Our results show that LPA and serum stimulation are not sufficient to cause endothelial cells along intact blood vessels adopt a lymphatic identity. The separation of endothelial cell identity was also maintained at the cell blood/lymphatic connection sites. The interpretation of our data remains limited by the unknown of whether phenotypic transition can be induced at all in the rat mesentery culture model. This unknown, however, can also be characteristic of the in vivo scenario and the contribution on this study is the demonstration that stimulations of blood endothelial cells in in vitro cell culture assays does do not have the same effect in cultured microvascular networks. Our results highlight an emerging and understudied area of lymphatic research and caution the translation of cell culture based endothelial plasticity studies to an adult network scenario, in which we speculate blood/lymphatic endothelial cell phenotypic transition requires a more complicated milieu of signals. Still, our understanding of lymphatic and blood endothelial cell identity in adult tissues is lacking and warrants future investigation.

## Conflict of Interest

None declared.
